# New-Onset Diabetes in Children during COVID-19: Clinical Case Report

**DOI:** 10.1155/2021/6654019

**Published:** 2021-02-10

**Authors:** M. Aabdi, A. Aarab, O. Es-Saad, K. Malki, H. Bkiyar, B. Housni

**Affiliations:** ^1^Anesthesiology and Intensive Care Unit Department, MOHAMMED VI University Hospital Center, Oujda, Morocco; ^2^Laboratory Unit Department, MOHAMMED VI University Hospital Center, Oujda, Morocco

## Abstract

**Introduction:**

Data of COVID-19 in newborns and children are limited, and clinical manifestations are nonspecific and might delay the diagnosis, which might lead to severe complications. In this clinical case, we will describe new-onset diabetes with consciousness impairment as an atypical revealing way of COVID-19.

**Case:**

A 3-year-old child presented to the Emergency Department with loss of consciousness (without fever), lethargy, and stupor. Clinical assessment on admission found an unconscious child with a pediatric Glasgow Coma Scale of 10/15 with no localizing signs or meningeal syndrome, polypneic of 35 breaths/min, pulse oximetry of 90%, with signs of overall dehydration: skin folds, sunken eyes, tachycardia of 160 beats/minute, and recoloring time superior at 3 seconds. Laboratory findings showed hyperleukocytosis of 16000/mm^3^, lymphopenia of 450/mm^3^, glycemia of 5 g/L with a correct ionogram : corrected natremia of 139 mmol/L, serum potassium of 4.5 mmol/L, glycosuria of 3+, ketonuria of 2+, and HbA1c of 10%, and COVID-19 RT-PCR came back positive.

**Conclusion:**

COVID-19 might be revealed with atypical symptoms including new-onset diabetes and diabetic ketoacidosis; therefore, clinicians must suspect it in children with blood glucose and HbA1c at the time of admission. This will help to manage patients with hyperglycemia early.

## 1. Introduction

From March 2020, Anesthesiology and Intensive Care Unit Department, Mohammed VI University Hospital Center, Oujda, Morocco, has been facing an unprecedented outbreak of coronavirus disease and spread of 2019 novel coronavirus or severe acute respiratory syndrome coronavirus 2 (SARS-CoV-2), which has now become a global pandemic [1, 2]. The disease caused by SARS-CoV-2 was termed “COVID-19,” and the World Health Organization declared the COVID-19 outbreak a public health emergency of international concern and officially was declared a pandemic on March 11, 2020 [2].

Data from the early months of 2020 suggest that there is a bidirectional relationship between COVID-19 and diabetes [3]. Diabetes is associated and is continuously suggested as a risk factor that contributes to the severity and mortality of COVID-19 [1]. Multiple forms of diabetes might be observed in patients with COVID-19 including new-onset diabetes and metabolic complications such as diabetic ketoacidosis and hyperosmolarity [4]. Nonetheless, data on new-onset type 1 diabetes during the COVID-19 pandemic, particularly in children, are limited [5]. In this article, we describe a clinical case of a 3-year-old child who presented to the Emergency Department (ED) in a ketoacidosis decompensation state, a recent diagnosis with COVID-19 infection, and who was retrospectively diagnosed with new-onset diabetes.

## 2. Clinical Case

A 3-year-old child presented to the ED with loss of consciousness (without fever), lethargy, and stupor. Clinical assessment on admission found an unconscious child with a pediatric Glasgow Coma Scale (GCS) of 10/15 with no localizing signs or meningeal syndrome, polypneic of 35 breaths/min, pulse oximetry of 90%, with signs of overall dehydration (skin folds), sunken eyes, tachycardia of 160 beats/minute, and recoloring time superior at 3 seconds.

Laboratory findings showed hyperleukocytosis of 16000 mm^3^, lymphopenia of 450 mm^3^, glycemia of 5 g/L with a correct ionogram : corrected natremia of 139 mmol/L, serum potassium of 4.5 mmol/L, glycosuria of 3+, ketonuria of 2+, and HbA1c of 10%.

**Figure 1:**
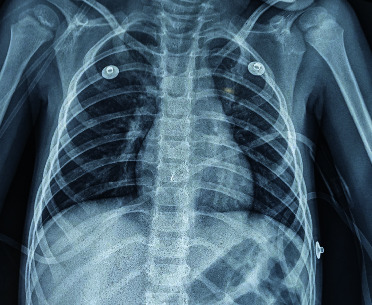
Chest X-ray showing an alveolar-interstitial syndrome.

**Figure 2:**
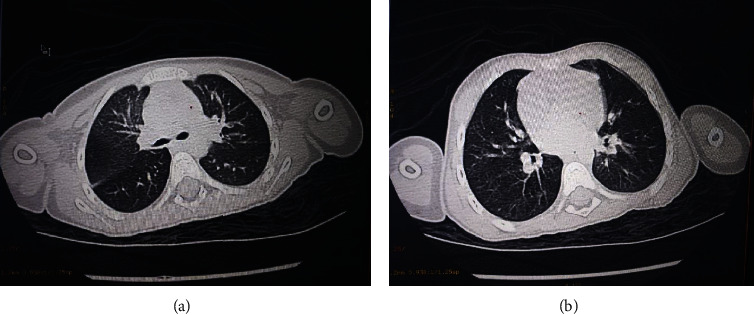
Chest CT scan showing images in favor of pneumopathy COVID-19 CO-RADS 4.

Brain computed tomography (CT) came back normal; the search for a primary infection site led us to carry out a chest X-ray showing an alveolar-interstitial syndrome (Figure 1), supplemented by a thoracic CT revealing a bilateral ground-glass opacification consistent with viral pneumopathy, with moderate impairment with a level of suspicion scored CO-RADS 4 (Figure 2). Given the epidemiological context, a COVID-19 RT-PCR came back positive.

Diabetic ketoacidosis (DKA) is the most common hyperglycemic emergency and is associated with increased morbidity and mortality. In children and adolescents with type 1 diabetes, DKA accounts for the most common cause of death. DKA is characterized by hyperglycemia, ketosis, and metabolic acidosis [6].

Management of DKA consisted of rehydration with Ringer's lactate 20 ml/kg initially and physiological serum as well as insulin therapy with a dose of 0.05 IU/kg/h along with monitoring of blood sugar levels and any complications. The prognosis was favorable with neurological, respiratory, and metabolic improvement; the child was transferred to the pediatric endocrinology department for further assessment.

## 3. Discussion

Coronaviruses are positive-strand ribonucleic, large viruses. Only two genera of the viruses can infect humans: a and b types, and they are called human coronaviruses (HCoVs) [7].

HCoVs are used to be mild phenotypes in humans because of their inconsequential pathogenic effect. However, two epidemics emerging in the early 21st century (severe acute respiratory syndrome (SARS) and Middle East respiratory syndrome (MERS)) with a high rate of morbidity and mortality changed the knowledge about this [8].

COVID-19 is generally more frequent among adults more than 15 years old with less confirmed cases among children [9]. However, the number of children infected has increased due to the pathogen detection campaigns and the lack of protective measures [10].

A report of a multicenter regional data from North West London of new-onset type 1 diabetes and DKA in children has estimated an increase of 80% of new type 1 diabetes cases during the COVID-19 pandemic and postulates that COVID-19 exposure contributed to the observed increase in cases by precipitating or accelerating type 1 diabetes onset [5]. A previous paper reported that children accounted for 5% of confirmed COVID-19 cases and presented with a milder disease symptom, and they have a better prognosis compared with adults, with very low mortality rate [7, 9]. But children with newly diagnosed diabetes, either it is new-onset or previously undiagnosed, tend to have increased levels of inflammatory markers and indicators of multiorgan injury leading to severe or critical illness of COVID-19 [11]. They also present severe hyperglycemic complications such as DKA and hyperosmolar hyperglycemic syndrome requiring high doses of insulin [12]. COVID-19 patients with newly diagnosed diabetes have higher mortality rate than those with known diabetes [11].

The pathophysiological mechanisms implicated with the increased frequency and severity of COVID-19 in people with diabetes are not yet elucidated, and several hypotheses have been elaborated to explain the link between COVID-19 and high rate and worst outcomes in diabetic people [2].

Diabetes is associated with an increased risk of infections, including viral ones due to defects in the innate immunity affecting phagocytosis, neutrophil chemotaxis, and cell-mediated immunity [1, 13]. Besides, diabetes and insulin resistance are associated with endothelial dysfunction and increased platelet aggregation and activation leading to the hypercoagulable prothrombotic state [13]. The latter associated with the low-grade chronic inflammation might promote the cytokine storm, a severe complication of COVID-19 characterized by excessive production of inflammatory cytokines [14]. Furthermore, COVID-19 spike protein gains entry to its target cells by using the angiotensin-converting enzyme 2 (ACE2) as the receptor, which has protective effects primarily regarding inflammation [1]. COVID-19 infection reduces ACE2 expression inducing cellular damage, hyperinflammation, and respiratory failure and can lead to a direct effect on *β-*cell function, which not only explains that diabetes might be a risk factor for a severe form of COVID-19 disease but also explains that infection could induce new-onset diabetes [1, 9, 15–18].

New-onset diabetes must be detected in all nondiabetic patients especially those at high risk for metabolic disease who have contracted the viral infection. Besides, most patients present multiple stresses with COVID-19 including respiratory failure and sepsis requiring intravenous infusion of insulin and fluid balance to control the glycemia levels [19]. In addition, potassium balance must be carefully considered in the context of insulin treatment, as hypokalemia is a common feature of COVID-19 and may be exacerbated after the introduction of insulin [19].

## 4. Conclusion

This clinical case supports the strong link between COVID-19 infection and diabetes. Therefore, physicians should consider this in all nondiabetic children and screen them at their admission with blood glucose and HbA1C for appropriate and early management.

## Data Availability

The data used to support the findings of this study are available from the corresponding author.
